# A Case of Von Hippel-Lindau Disease With Recurrence of Paraganglioma and No Other Associated Symptoms: The Importance of Genetic Testing and Establishing Follow-Up Policies

**DOI:** 10.7759/cureus.50484

**Published:** 2023-12-13

**Authors:** Naoki Okada, Akihiro Shioya, Sumihito Togi, Hiroki Ura, Yo Niida

**Affiliations:** 1 Department of Pediatrics, Kanazawa Medical University, Uchinada, JPN; 2 Department of Pathology and Laboratory Medicine, Kanazawa Medical University, Uchinada, JPN; 3 Center for Clinical Genomics, Kanazawa Medical University Hospital, Uchinada, JPN; 4 Department of Advanced Medicine, Division of Genomic Medicine, Medical Research Institute, Kanazawa Medical University, Uchinada, JPN

**Keywords:** next-generation sequencing, molecular genetic testing, hereditary paraganglioma-pheochromocytoma syndromes, paraganglioma, von hippel-lindau disease

## Abstract

Pheochromocytoma and paraganglioma (PPGL) are rare neuroendocrine tumors. Catecholamine production by the tumors leads to high blood pressure. Although most PPGLs are benign, some have metastatic potential. Almost half of PPGLs are caused by germline mutations, and the causative genes are diverse. Von Hippel-Lindau disease (VHL) is an autosomal dominant multisystem tumor predisposition syndrome characterized by central nervous system and retinal hemangioblastomas, clear cell renal cell carcinoma, pancreatic neuroendocrine tumors, and PPGLs. Sometimes VHL presents only as paraganglioma (PGL), making its diagnosis difficult. A male child aged five years and one month was found to have isolated catecholamine-producing PGL in the right renal hilum during evaluation for hypertension. The patient was completely cured by tumor resection, and somatic mutation testing of the tumor revealed no abnormalities. At the age of nine years and 11 months, the patient had a recurrence of PGL in the left border of the abdominal aorta. Comprehensive germline genetic testing was performed and revealed a pathologic missense variant NM_000551.4:c.482G>A p.(Arg161Gln) in the* VHL* gene. This variant showed loss of heterozygosity in both primary and recurrent tumors by Sanger sequencing, and DNA microarray analysis revealed a monosomy of the entire chromosome 3 where *VHL* is located. Arg161Gln has been previously reported in several other VHL families, and the symptoms were diverse beyond PPGLs. This case demonstrates the importance of genetic diagnosis with VHL in mind. It was also recognized that this patient needed to be followed for symptoms of VHL other than PGL.

## Introduction

Pheochromocytoma (PHEO) and paraganglioma (PGL) are rare neuroendocrine tumors that arise from chromaffin cells. PHEOs arise from the adrenal medulla, whereas PGLs arise from the neural crest localized outside the adrenal gland [1]. Since the two tumor types cannot be differentiated on the basis of histologic findings, anatomical location is used to distinguish between them, and the overall incidence is approximately 0.6 cases per 100,000 person-years [2]. Although most pheochromocytoma and paraganglioma (PPGL) are benign, approximately 10% have metastatic potential. Approximately 40% of PPGL cases carry germline mutations [1,3]. More than 20 genes are known to cause inherited PPGL [1], and approximately 20% of these are caused by pathogenic germline variants in succinate dehydrogenase complex (*SDHx*), *TMEM127,* or *MAX* genes [4]. Less frequently, mutations in the genes responsible for Von Hippel Lindau disease (VHL), multiple endocrine neoplasia type 2 (MEN2) and neurofibromatosis type 1 (NF1) are also found in patients with hereditary PPGL [5]. Most patients with metastatic PPGL are sporadic, whereas in patients with inherited PPGL, metastatic tumors caused by *SDHB *mutations account for up to 43% of cases, followed by *VHL*, *SDHD*, and *NF1* mutations [2].

VHL (OMIM #193300) is an autosomal dominant multisystemic tumor predisposition syndrome characterized by benign and malignant tumors, including PPGL, central nervous system and retinal hemangioblastomas, clear cell renal cell carcinoma (RCC), pancreatic neuroendocrine tumors, endolymphatic sac tumors, and epididymal and broad ligament cystadenomas, as well as renal and pancreatic cysts [6]. The incidence of VHL is estimated to be one in 36,000. The lifetime penetrance is close to 100% by the age of 75 years [6]. Clinically, VHL is divided into two major types: type 1, which does not involve the PPGL, and type 2, which does involve the PPGL. Type 2 is further subdivided into type 2a, which is associated with central nervous system and retinal hemangioblastoma (CNB/RB) but not RCC, type 2b, which is associated with both CNB/RB and RCC, and type 2c, which is associated with neither [7]. Although little is known about the proportion of each type, the lifetime risk of developing PPLG in VHL patients is estimated to be 10-25%, which is consistent with the frequency of type 2 [6]. According to the results of a nationwide survey in Japan, 62 (15%) of 409 registered VHL patients developed PPGL, of which 31 were type 2A, 20 were type 2B, and 11 were type 2C [8]. The frequency of type 2C is equivalent to 2.7% of all VHL patients, which is an indication that it is a very rare phenotype. Accordingly, PPGL itself is a rare tumor, and VHL is rarely found in patients with PPGL alone, making diagnosis extremely difficult. Here we report a patient with VHL who developed PGL at the age of five years and relapsed four years later without any symptoms other than PGL. There will be a discussion of the importance of genetic diagnosis and appropriate follow-up.

## Case presentation

A male child, aged five years and one month, was admitted to our hospital for evaluation of hypertension. His height, weight, and blood pressure were 106 cm (-0.2 SD), 15.5 kg (-0.9 SD), and 154/114 mmHg, respectively. Headaches and excessive sweating were reported. Although there was no family history of PPGL, his paternal grandfather had renal cancer. Urinalysis, complete blood count, and blood biochemistry showed no abnormalities. Endocrinologic tests showed normal thyroid function and elevated catecholamines as plasma norepinephrine 1529 pg/ml, urinary norepinephrine 323.9 μg/day, urinary normetanephrine 1.61 μg/mg·Cr (Table 1).

**Table 1:** Catecholamine levels in blood and urine

Catecholamines	Reference values	Initial onset	Recurrence
Plasma			
Dopamine	< 30 pg/ml	10	≤ 5
Adrenaline	≤ 100 pg/ml	67	80
Noradrenaline	100-450 pg/ml	1529	488
Urine			
Adrenaline	3.4-26.9 μg/day	11.5	8.0
Noradrenaline	48.6-168 μg/day	323.9	222.4
Metanephrine	0.04-0.19 mg/day	0.08	0.12
Normetanephrine	0.09-0.33 mg/day	0.76	0.63

**Figure 1:**
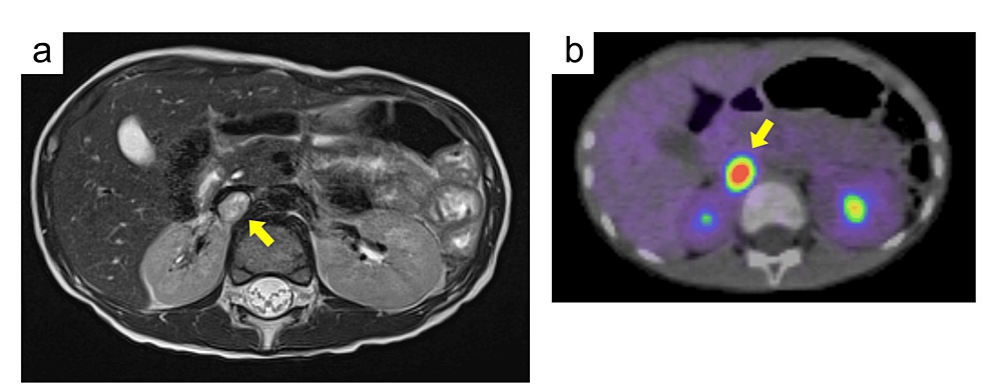
Imaging studies of the patient at initial onset Axial abdominal MRI T2-weighted images (a) and 18F-FDG-PET (b). Tumors are indicated by yellow arrows. FDG: F-18 fluorodeoxyglucose; PET: positron emission tomography

Abdominal MRI showed a 1.4 cm tumor with speckled high signal intensity on T2-weighted images near the right renal hilum (Figure 1a). 18F-fluorodeoxyglucose (FDG) positron emission tomography (PET) showed localized accumulation near the right renal hilum with a maximum standardized uptake value (SUVmax) of 8.2 (cut-off > 3.0), but no other uptake consistent with distant metastases was observed (Figure 1b). There was no accumulation at the lesion on 123I-metaiodobenzylguanidine (MIBG) scintigraphy. The preoperative diagnosis was a noradrenaline-producing retroperitoneal PGL. Hypertension was treated with oral doxazosin and enalapril maleate, and tumor resection was performed at the age of five years and four months. Antihypertensive therapy was discontinued immediately after surgery, the postoperative course was uneventful, and the patient recovered without complications.

**Figure 2:**
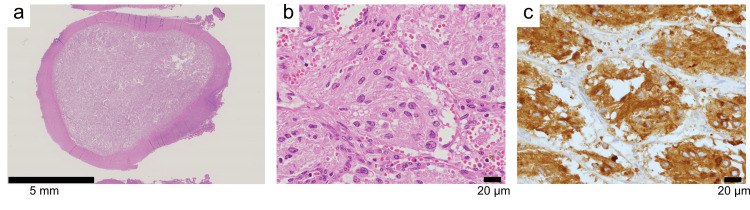
Pathological findings of initial PGL Hematoxylin and eosin staining (a, b);  Immunohistochemical staining of chromogranin A (c). PGL: paraganglioma

The removed tumor was 1.5 cm in size with a hard capsule and no capsule rupture was observed (Figure 2a). Tumor growth exhibited the so-called zellballen pattern, consisting of well-developed short spindle-shaped to polygonal tumor cells showing nested growth with an intervening stromal component of fibrovascular tissue and peripheral sustentacular cells (Figure 2b) [2]. Immunohistochemically, the tumor cells were diffusely stained by chromogranin A (Figure 2c), and the tumor was confirmed as PGL. The Ki-67 positive cell rate was 4%. The grading of adrenal PPGL (GAPP) score was 4 points, which predicts metastatic potential based on histopathologic evaluation and the type of catecholamine produced, 0-2 low risk, 3-6 intermediate risk, and 7-10 high risk [9]. Tumor tissue DNA was tested for succinate dehydrogenase (SDH) genes (*SDHB*, *SDHAF2*, *SDHB*, *SDHC*, and *SDHD*) by targeted DNA sequencing, but no pathogenic variants were detected. Antihypertensive medication was discontinued immediately after surgery and no hypertension was observed. Endocrinological tests on postoperative day 14 showed a decrease in urinary noradrenaline to 34.6 μg/day and urinary normetanephrine to 0.08 mg/day. Thereafter, we periodically evaluated blood and urine catecholamines and performed follow-up observations using 18F-FDG PET and abdominal MRI.

**Figure 3:**
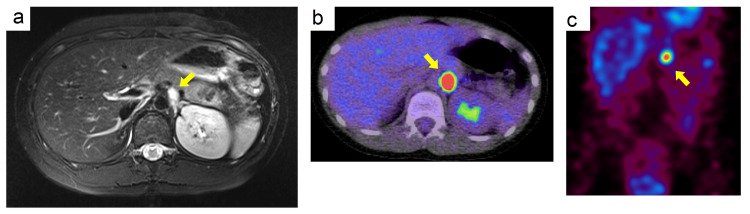
Imaging studies of the patient at recurrence Axial abdominal MRI T2-weighted images (a), 18F-FDG-PET (b), and coronal 123I-MIBG scintigraphy (c). Tumors are indicated by yellow arrows. FDG: F-18 fluorodeoxyglucose; PET: positron emission tomography; MIBG: meta-iodobenzylguanidine

At four years and seven months after surgery (when the patient was nine years and 11 months), there were no subjective symptoms such as headache or excessive sweating as observed at the time of initial onset, and blood pressure was normal at 111/83 mmHg, but Abdominal MRI showed a 1.2 cm tumor with high signal intensity on T2-weighted images at the same site (Figure 3a), and urinary normetanephrine level was elevated to 1.11 μg/mg·Cr (Table 1). 18F-FDG-PET showed an accumulation of SUVmax 19.5 localized to the left border of the abdominal aorta (Figure 3b) and there was an accumulation on 123I- MIBG scintigraphy (Figure 3c). It was diagnosed as a recurrence of PGL.

**Figure 4:**
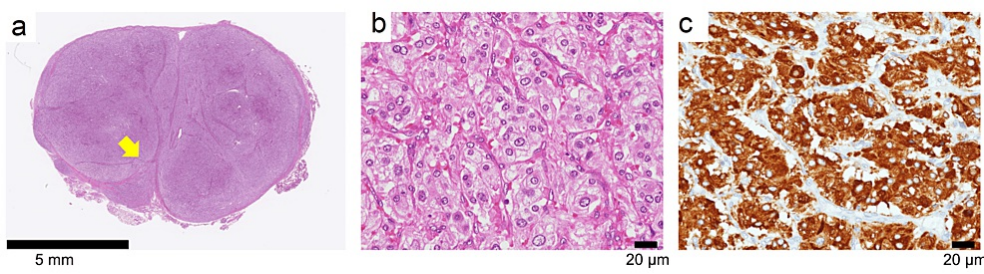
Pathological findings of recurrent PGL Hematoxylin and eosin staining (a, b). Rupture of the capsule was observed in recurrent PGL (yellow arrow in a). Immunohistochemical staining of chromogranin A (c). PGL: paraganglioma

The tumor was resected at the age of 10 years zero months after the same antihypertensive therapy as at the time of initial PGL. The size of the resected tumor was 15 mm. In contrast to the first surgery, some rupture of the capsule was observed (Figure 4a). Polygonal tumor cells were observed growing in a zellballen pattern, similar to the initial tumor (Figure 4b). The tumor cells were chromogranin A positive (Figure 4c), confirming the recurrence of the PGL. The Ki-67 positive cell rate was 5% and the GAPP score was 5 points. Two months after surgery, urinary normetanephrine decreased to 0.25 μg/mg·Cre, and no accumulation was observed on 18F-FDG-PET. The patient was subsequently followed with catecholamines and imaging studies and is currently 11 years and one month old and in remission.

**Figure 5:**
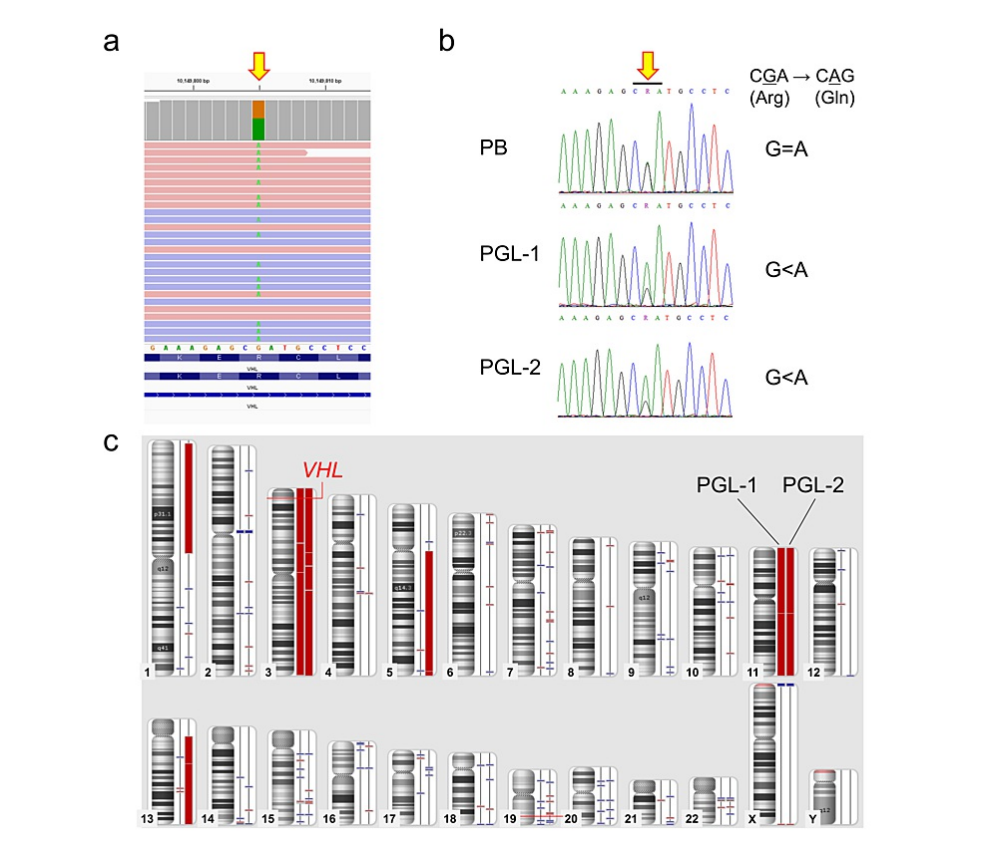
Genetic analysis. A missense variant in *VHL*, NM_000551.4:c.482G>A p.(Arg161Gln), was detected by next-generation sequencing (a), and Sanger sequencing revealed loss of heterozygosity in both PGLs (b). DNA microarray showed complete monosomy of chromosome 3 in both PGLs, and the recurrent PGL showed a pattern of chromosomal abnormalities derived from the initial PGL. PGL: paraganglioma

Considering the possibility of hereditary PPGL syndrome, we provided genetic counseling to the patient and parents, obtained their consent, and performed genetic testing using the TruSight One Expanded panel (Illumina, Inc., San Diego, California, United States). Shortly, DNA was extracted from peripheral blood using a standard method. The library was prepared from 50 ng of DNA according to the manufacturer's recommended protocol, and a 12.5 pM library was sequenced on an Illumina MiSeq system (2 × 250 cycles) following the standard Illumina protocol. Data analysis was conducted as previously reported [10]. In short, haplotype variant calling was performed using HaplotypeCaller version 4.0.6.0 (GATK (Genome Analysis Toolkit); Broad Institute, Cambridge, Massachusetts, United States) [11], and functional classification of variants was performed using SnpEff (version 4.3t) [12]. The Integrative Genomic Viewer (IGV version 2.4.13) was used for visualization [13]. The previously reported pathogenic missense variant was detected in *VHL*, NM_000551.4:c.482G>A p.(Arg161Gln) (Figure 5a). The variant was confirmed by Sanger sequencing using the VHL exon 3 specific primer set (VHL_Ex3-F: 5'-TACAGGTAGTTGTTGGCAAAGC-3' and VHL_Ex3-R: 5'-GAAACTAAGGAAGGAACCAGTCC-3', product size 360 bp) and BigDye Terminator v3.1 cycle sequencing kit on the ABI PRISM 3100xl genetic analyzer (Thermo Fisher Scientific Inc., Waltham, Massachusetts, United States). It turned out that this patient had von Hippel-Lindau disease, and the PGL was a symptom of that disease. In PGLs, the peak of the wild-type allele decreased in both tumors, suggesting that loss of heterozygosity of the *VHL* gene occurred within the tumors (Figure 5b). Structural chromosomal aberration analysis was performed by DNA microarray (OncoScan™ CNV Assay, Thermo Fisher Scientific Inc.), and both tumors showed monosomy for the entire chromosome 3 (Figure 5c). In the PGL at the time of recurrence, partial monosomies were observed on chromosomes 1, 5, and 13, which were not observed in the PGL at the time of initial onset. On the other hand, monosomy of chromosomes 3 and 11 was common to both tumors, suggesting that the original tumor had recurred with additional chromosomal abnormalities.

After the diagnosis of VHL, a systemic screening was performed to evaluate for other lesions of VHL. However, no abnormalities were found on brain MRI or fundus examination, and no lesions other than the PGL were found in the patient.

## Discussion

Our patient was diagnosed with VHL type 2c. The only symptom was PGL, which is considered a rare case of VHL. The type of VHL pathogenic variant has been shown to account for differences in PPGL risk. There is a strong genotype-phenotype correlation, and protein-truncating variants have been identified in individuals with type 1, whereas missense variants have been associated with type 2 [6]. The risk of CNB/RB and RCC in affected individuals may reflect the ability of the variant protein to regulate the hypoxia-inducible factor (HIF) pathway. Higher levels of HIF expression appear to be associated with a lower risk of CNB/RB and RCC [14]. The two most common type 2C-associated *VHL* missense mutations are the amino acid substitutions Val84Leu and Leu188Val [6]. There are also reports of Gly93Ser with PHEO [15], and Leu163Phe with PGL [16]. The *VHL* missense variant Arg161Gln (R161Q) detected in our patient has been previously reported in another family. Qi et al. reported a family with VHL R161Q variant and all three patients in the family had no symptoms other than PHEO consistent with type 2c [17]. Santarpia et al. reported a patient with type 2c VHL, who had bilateral PHEOs, multiple PGLs, and an extra-axial supratentorial frontal meningioma [18]. Iida et al. reported a variety of phenotypes in a family with the R161Q variant. The proband has VHL type 2A with PHEO and retinal hemangioblastoma (RB) [19]. Another family member also developed a large PHEO and RB. In addition, another patient developed neuroendocrine tumors of the pancreas without a PHEO. These cases show that the symptoms associated with R161Q are diverse and not necessarily limited to type 2c. In our patient in the current case, it was not confirmed whether the variant was inherited or a de novo mutation because the parents were reluctant to undergo genetic testing; and the relationship with the paternal grandfather's renal cancer cannot be denied. Follow-up is required to monitor not only for PGL recurrence but also for other VHL lesions to develop.

Deciding when and to what extent to perform genetic testing for PPGL is difficult. Sixty percent of PPGL is not hereditary, and hereditary PPGL genes are diverse. In the current case, the SDHx genes were tested in the PGL at the time of onset to assess the risk of tumor metastasis, but no mutation was found, so germline genetic testing was not performed. At the time of the relapse, the patient had germline genetic testing and the diagnosis was VHL. According to an Endocrine Society clinical practice guideline, all patients with PPGLs should be engaged in shared decision-making for genetic testing [20]. This guideline also provides an algorithm for determining which genes to test. When the patient is non-syndromic, has no metastases, and has a noradrenergic tumor in the extra-adrenal gland,* SDHB*, *SDHD*, *SDHC*, *VHL*, and *MAX* are tested. In the present case, there were no symptoms other than PGL, so we could not initially diagnose it as syndromic (VHL), but according to the algorithm above, *VHL* is also a first-line target gene. In addition, according to another guideline for genetic testing for inherited PPGL by Muth et al., *FH*, *NF1*, *RET*, *SDHB*, *SDHD*, and *VHL* should be tested as a minimum, and the addition of MEN1, SDHA, SDHAF2, SDHC, TMEM127, and MAX is recommended [4]. Again, *VHL* is listed as one of the genes that should be tested. RB and PHEO are also the lesions that occur in the youngest patients with VHL, as the minimum/average age of 0/25 and 2/27, respectively, makes *VHL* genetic testing particularly important in pediatric patients with PPGLs.

## Conclusions

Although rare, it is necessary to consider VHL in the differential diagnosis of isolated PGL. PGL may be the only symptom of VHL. Genetic diagnosis is important for proper diagnosis and medical intervention, especially in pediatric patients. If germline genetic testing for PLG is performed, it must include VHL. In the present case, a definitive diagnosis was delayed because the initial genetic testing was performed for somatic mutations of the SDHx genes in the tumor. The development of PGL tends to correlate with VHL missense mutations, but other VHL lesions may be highly variable among patients. A variety of phenotypes have been reported for R161Q, and it is necessary to follow up on this patient, keeping in mind the occurrence of VHL lesions other than PGL.
